# Understanding the mechanism of Pd-catalyzed allylic substitution of the cyclic difluorinated carbonates

**DOI:** 10.3762/bjoc.4.18

**Published:** 2008-05-27

**Authors:** Jun Xu, Xiao-Long Qiu, Feng-Ling Qing

**Affiliations:** 1 Key Laboratory of Organofluorine Chemistry, Shanghai Institute of Organic Chemistry, Chinese Academy of Sciences, 354 Fenglin Lu, Shanghai 200032, China; 2 College of Chemistry, Chemical Engineering and Biotechnology, Donghua University, 2999 North Renmin Lu, Shanghai 201620, China

## Abstract

We present a mechanistic investigation of Pd-catalyzed allylic substitution of cyclic *gem*-difluorinated carbonates **1** and **4**, previously employed in the synthesis of 3',3'-difluoro-2'-hydroxymethyl-4',5'-unsaturated carbocyclic nucleosides in 17 steps. The substitution features a reversal of regioselectivity caused by fluorine.

## Background

Carbocyclic nucleosides (CNAs), in which the furanose oxygen atoms of the 4'-oxonucleosides are substituted by CH_2_, have received considerable attention because they exhibit greater metabolic stability toward nucleoside phosphorylases and higher lipophilicity, two properties that are potentially beneficial in terms of increased in vivo half life, oral efficiency and cell wall penetration [1–2]. Based on CNA skeletons, 1,2-disubstituted carbocyclic nucleosides (OTCs) recently attracted more and more attention [3–9], especially after De Clercq *et al.* found that some OTCs showed moderate to good activity against murine leukemia cells L1210/0, human T-lymphocyte cells Molt4/C8, and CEM/0 via topological substructural approach to molecular design (TOSS-MODE) [10]. As part of our ongoing and continual efforts to prepare potential bioactive fluorinated nucleosides, our group recently described the stereoselective synthesis of 3',3'-difluoro-4',5'-unsaturated OTCs **2–3** and **5** [11]. The whole synthesis highlighted the stereoselective Reformatskii-Claisen rearrangement, ring-closing metathesis (RCM), and Pd-catalyzed allylic substitution, in which the regioselectivity was reversed from that of nonfluorinated substrates. This reversed regioselectivity caused by fluorine interests us greatly. Herein, we present a mechanistic investigation of Pd-catalyzed allylic substitution of cyclic *gem*-difluorinated carbonates.

## Results and Discussion

**Scheme 1:**
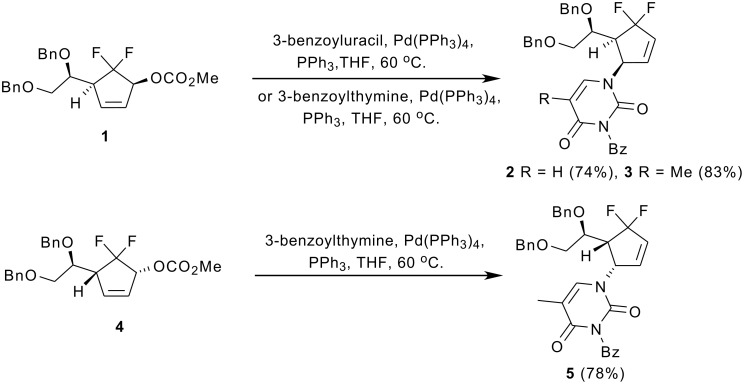
Pd-catalyzed allylic substitution of the *gem*-difluorinated allylic carbonates **1** and **4**.

On installation of pyrimidine bases into the *gem*-difluorinated allylic carbonates **1** and **4**, our group found that the γ-substitution products **2,** **3** and **5** were surprisingly generated exclusively in good yields, respectively, when the compounds **1** and **4** reacted with suitably protected nucleobases 3-benzoyluracil and 3-benzoylthymine in the presence of a catalytic amount of Pd(PPh_3_)_4_ at 60 °C in THF (Scheme 1) [11]. The exclusive regioselectivities of Pd-catalyzed allylic alkylation (Pd-AA) reactions were very interesting. Although Konno *et al.* have reported that the electron-withdrawing fluoroalkyl groups would alter the regioselectivities of acyclic allylic alkylation compared with their non-fluorinated counterparts [12–17], their reactions mostly concerned the Pd-catalyzed regio- and stereoselective formate reduction of fluorine-containing allylic mesylates. To the best of our knowledge, the effect of *gem*-difluoromethylene group on Pd-catalyzed cyclic allylic substitution has never been addressed so far. The regioselectivity was totally different from those of nonfluorinated substrates [18].

Unexpected and specific regioselectivity of Pd-catalytic asymmetric reactions of the *gem*-difluorinated allylic intermediates **1** and **4** prompted us to investigate further the mechanism of these reactions. Currently, one of the most direct tactics for mechanistic investigation of Pd-AA reaction was built on the analysis of crystal structure and ^13^C NMR spectroscopy of Pd-π-allyl complex [19–20]. The orientation of attack of nucleophiles on the Pd-π-allyl complex could be illustrated via examining the ^13^C NMR chemical shifts of three carbon atoms attached to the palladium. According to the model of DeShong *et al.* [21], it was anticipated that a symmetrical Pd-π-allyl complex should be temporarily generated once the compounds **1** or **4** were treated with palladium catalyst. Thus, α-substitution products should be afforded considering the steric effects. However, only γ-substitution products **2–3** and **5** were isolated in our case, which, in our opinion, resulted from the specific electron-withdrawing property of the *gem*-difluoromethylene group. To further validate our hypothesis and the proposed model of DeShong *et al.*, we decided to explore the crystal structure and ^13^C NMR of the corresponding Pd-π-allyl complex.

**Scheme 2:**
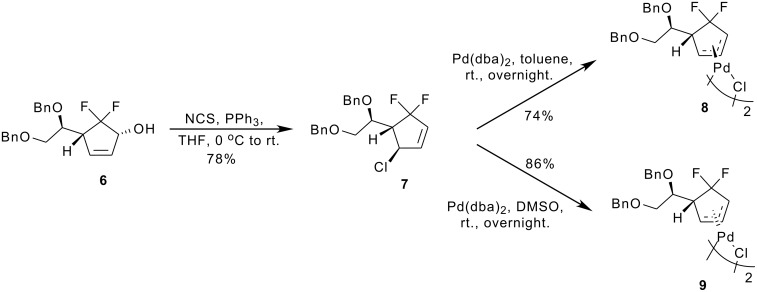
Synthesis of *trans*-*trans* dimer **8** and *syn*-*syn* dimer **9**.

In 2000, Bäckvall and co-workers investigated the X-ray structures for *cis* and *trans* isomers of [(1,2,3-η)-4-acetoxycyclohex-2-enyl]palladium chloride dimers [22]. They found that the X-ray structure of *trans*-*trans* dimer displayed asymmetric allyl-palladium bonding where the Pd-C3 bond was shorter than the Pd-C1 bond. Their study provided the first direct evidence for the presence of electronic interaction in the Pd-catalytic asymmetric allylic reactions of 1-acetoxy-4-chloro-2-cyclohexene. Based on their study, we first converted the precursor compound **6** to the γ-chloro-subsituted compound **7** in 78% yield by treatment with NCS / PPh_3_ in THF at 0 °C (Scheme 2). Using the stereodivergent method of Kurosawa *et al.* [23], *trans*-*trans* dimer **8** and *cis*-*cis* dimer **9** were prepared in 74% and 86% yield by treatment of the chloride **7** with Pd(dba)_2_ using toluene and DMSO as the solvent, respectively.

**Figure 1:**
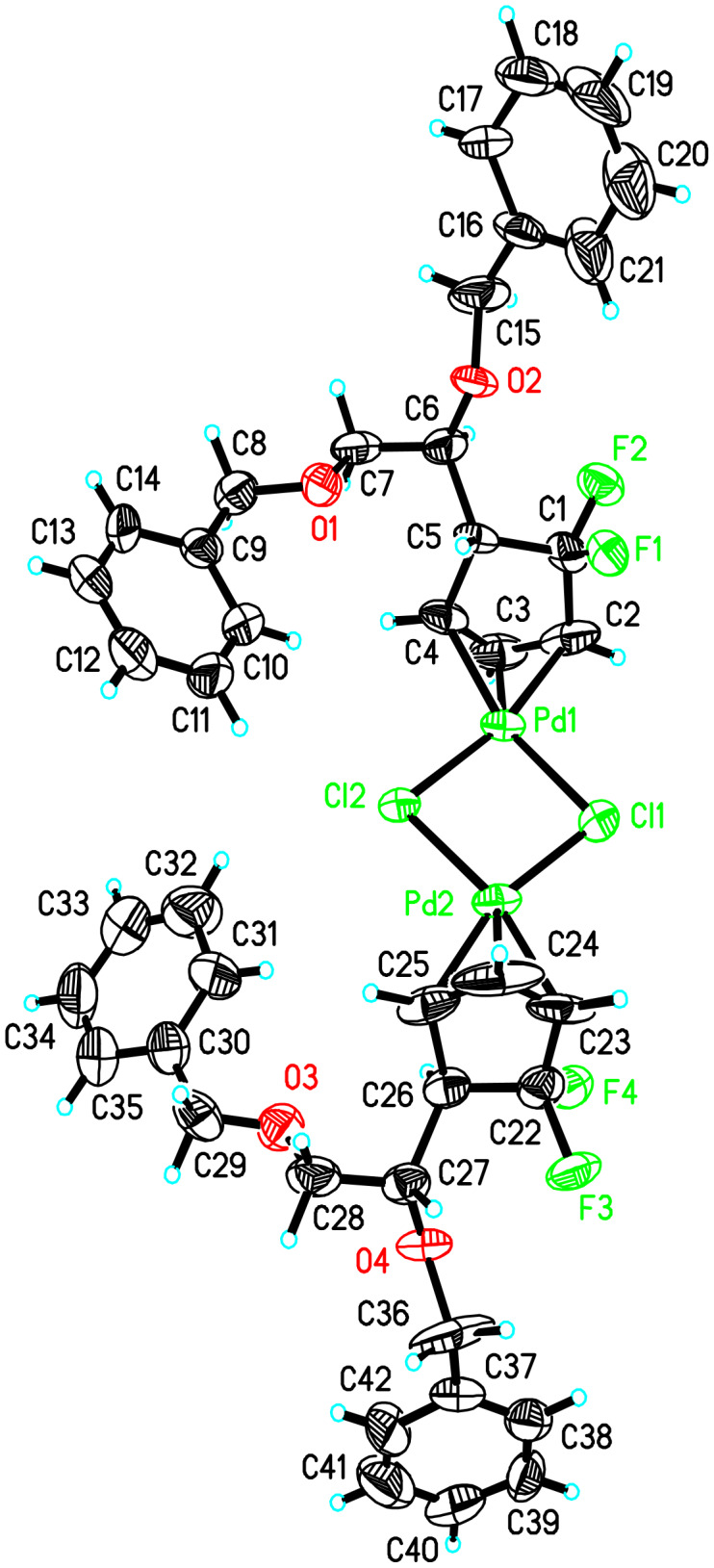
X-ray structure of *trans*-*trans* dimer **8**.

To our delight, the crystal of *trans*-*trans* dimer **8** was suitable for X-ray analysis (Figure 1). It was clear that 1,2-bis(benzyloxy)ethyl moieties in dimer **8** occupied the positions *trans* to the corresponding palladium atoms. Also obvious was that allyl-palladium bonding in *trans*-*trans* dimer **8** was almost symmetric: within experimental error, there was not much difference between the Pd1-C2 bond length 2.105 Å (11) and Pd1-C4 bond length 2.118 Å (9), and between the Pd1-C4-C3 bond angle 68.9° (6) and Pd1-C2-C3 bond angle 69.2° (7) (Table 1). Thus, as expected from the proposed model [21], the symmetrical Pd-π-allyl complex was generated. According to the X-ray structure of the *trans*-*trans* dimer **8**, it was clear that C4 position was more shielded than the C2 position, which should guide the attack of nucleophiles from the C2 position. However, we only isolated the product resulting from the attack of nucleophiles from C4 position, which, in our opinion, was totally attributed to the strong electron-withdrawing property of neighboring *gem*-difluoromethylene group. ^13^C NMR spectroscopy of the *trans*-*trans* dimer **8** unambiguously demonstrated that C2 (t, δ = 73.2) experienced higher field than C4 (s, δ = 81.4), which was also ascribed to the strong electron-withdrawing property of the CF_2_ group. Taking all the above analysis together, it seems that the CF_2_ group's strong electron-withdrawing property leads to a higher density of positive charge at the C4 position of the π-allyl palladium, which predominantly promotes attack of the nucleophile on C4 even when the C4 position is more hindered than the C2 position.

**Table 1:** Selected bond lengths (Å), bond angle (^o^) and chemical shifts of ^13^C NMR spectroscopy of the *trans*-*trans* dimer **8**.

	bond length	bond angle	^13^C NMR

Pd1-C2	2.105 Å (11)	–––––	–––––
Pd1-C4	2.118 Å (9)	–––––	–––––
Pd1-C4-C3	–––––	68.9° (6)	–––––
Pd1-C2-C3	–––––	69.2° (7)	–––––
C2	–––––	–––––	73.1 (t, *J* = 19.3 Hz)
C4	–––––	–––––	81.4 (s)

**Scheme 3:**
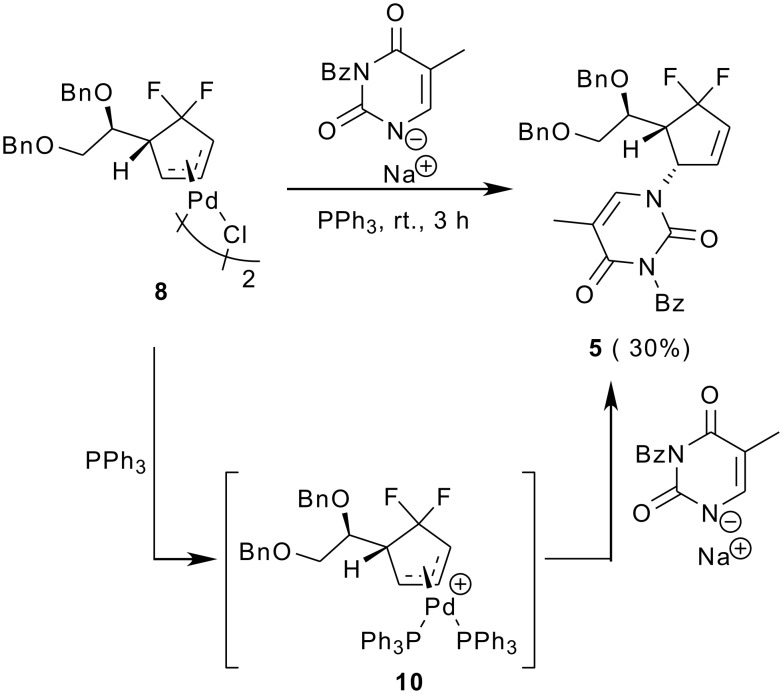
Reaction of *trans*-*trans* dimer **8** with 3-benzoylthymine sodium / PPh_3_.

**Scheme 4:**
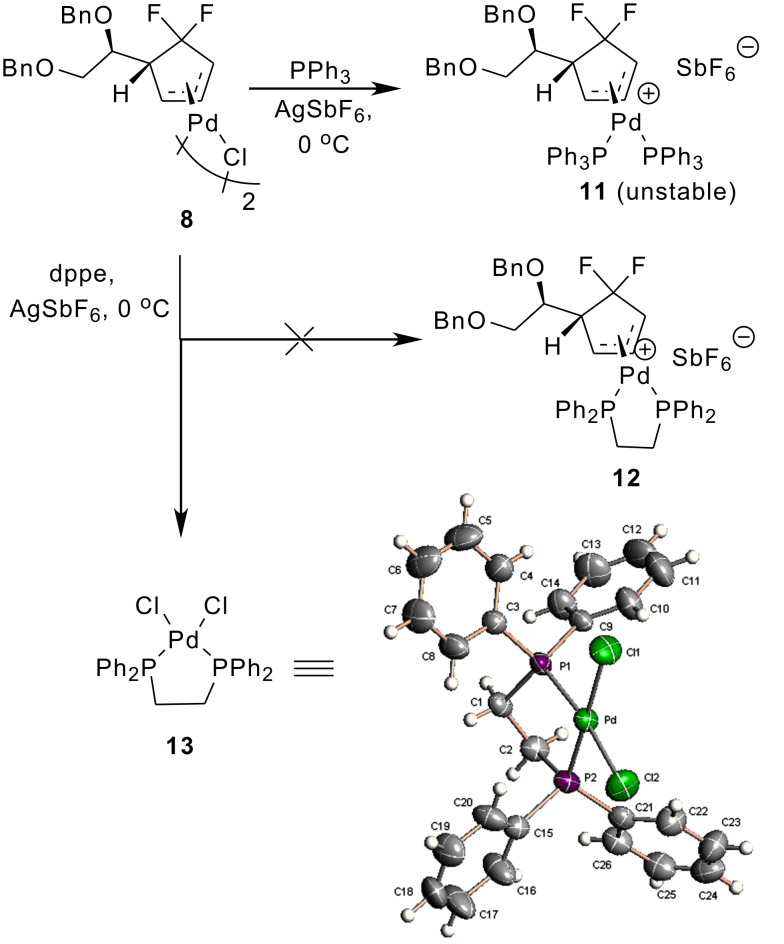
Reaction of *trans*-*trans* dimer **8** with PPh_3_ / AgSbF_6_.

Interestingly, we also found that treatment of the *trans*-*trans* dimer **8** with PPh_3_ / 3-benzoylthymine sodium at rt provided the nucleoside analogue **5** in 30% yield. No product was detected when PPh_3_ was absent (Scheme 3). Thus, we think that the reaction occurred involving the monopalladium complex **10**. The complex **10** resulted from the depolymerization of the dimer **8** caused by PPh_3_. With this thought in mind, we attempted to isolate and characterize the monopalladium complex. Unfortunately, we found that exposure of the *trans*-*trans* dimer **8** to PPh_3_ / AgSbF_6_ afforded the monopalladium complex **11**, which was too unstable to be isolated (Scheme 4). Neither did substitution of PPh_3_ by dppe deliver our desired complex **12** but gave instead the palladium complex **13**, whose structure was confirmed by X-ray structure analysis. In our opinion, the reason we failed to isolate the monopalladium complex was also due to the presence of *gem*-difluoromethylene group. That is, in the absence of nucleophiles, the dimer **8** tended to lose the cyclopentanyl ligand upon treatment with PPh_3_ or dppe, because the CF_2_ group made the Pd-π-allyl too electron-deficient. That was why we could isolate the palladium complex **13** as the only product.

In conclusion, we have investigated the reaction mechanism of Pd-catalyzed allylic substitution of cyclic *gem*-difluorinated intermediates in detail via the crystal structure and ^13^C NMR spectroscopy of the Pd-π-allyl complex. We found that the Pd-catalyzed reactions of cyclic *gem*-difluorinated allylic carbonates **1** and **4** proceeded via the symmetric Pd-π-allyl bonding and highly regioselective γ-substitution resulted from the neighboring *gem*-difluoromethylene group. We propose that the present work opens a new avenue for the further insight into the Pd-catalyzed allylic substitution reactions.

## Supporting Information

File 1 Experimental Section and Characterization Data of Compounds
