# Supplementary material for: Understanding the mechanism of Pd-catalyzed allylic substitution of the cyclic difluorinated carbonates
Source: Beilstein J Org Chem. 2008 May 27;4:18. doi: 10.3762/bjoc.4.18 (PMC2486488; doi:10.3762/bjoc.4.18)
Supplement: File 1 — Experimental Section and Characterization Data of Compounds [file Beilstein_J_Org_Chem-04-18-s001.doc]

# SUPPORTING INFORMATION

### Understanding the mechanism of Pd-catalyzed allylic substitution of the cyclic difluorinated carbonates

Jun Xu^1^, Xiao-Long Qiu^1^, and Feng-Ling Qing* ^1, 2^

Address: ^1^Key Laboratory of Organofluorine Chemistry, Shanghai Institute of Organic Chemistry, Chinese Academy of Sciences, 354 Fenglin Lu, Shanghai 200032, China and ^2^College of Chemistry, Chemical Engineering and biotechnology, Donghua University, 2999 North Renmin Lu, Shanghai 201620, China

Email: Jun Xu - xujun@mail.sioc.ac.cn;

Xiao-Long Qiu - chemqiu@yahoo.com.cn;

Feng-Ling Qing***** - [flq@mail.sioc.ac.cn](mailto:flq@mail.sioc.ac.cn)

** Corresponding author*

**Experimental Section**

*General information*: All reagents were used as received from commercial sources, unless specified otherwise, or prepared as described in the literature. Tetrahydrofuran and toluene were distilled from sodium and benzophenone immediately before use. ^1^H NMR and ^13^C NMR spectra were recorded on a Bruker AM-300 spectrometer or Bruker AM-400. ^19^F NMR was recorded on a Bruker AM-300 spectrometer (FCCl_3_ as outside standard and low field is positive). Chemical shifts (δ) are reported in ppm, and coupling constants (*J*) are in Hz. The following abbreviations were used to explain the multiplicities: s = singlet, d =doublet, t =triplet, q =quartet, m = multiplet. Compound **6** was prepared using our published procedures (*Org. Lett.* **2007,** *9,* 5437-5440).

**{(*S*)-1-[(1*R*,5*R*)-5-Chloro-2,2-difluorocyclopent-3-enyl]ethane-1,2-diyl}bis(oxy)bis(methylene)dibenzene (7):** NCS (76 mg, 0.56 mmol) was dissolved in THF (2 mL) and PPh_3_ (150 mg, 0.57 mmol) was added. The mixture was stirred at rt for 20 min and then cooled to 0 ^o^C and then a solution of **6** (137 mg, 0.38 mmol) in THF (2 ml) was added. The reaction mixture was warmed to rt and stirred overnight. After removing the solvent in vacuo, the residue was purified by flash chromatography (petroleum ether : ethyl acetate = 15 : 1) on silica gel to give **7** (112 mg) in 78% yield as clear oil. [α]D 26 = −83.31 (*c* 0.80, CHCl_3_); ^1^H NMR (300 MHz, CDCl_3_) δ 7.37-7.24 (m, 10H), 6.25 (d, *J* = 5.7 Hz, 1H), 6.02 (d, *J* = 5.7 Hz, 1H), 4.83-4.82 (m, 1H), 4.71 (dd, *J* = 40.2 Hz, *J* = 11.7 Hz, 2H), 4.58 (s, 2H), 4.00-3.94 (m, 1H), 3.87-3.76 (m, 2H), 2.99-2.86 (m, 1H); ^13^C NMR (75.5 MHz, CDCl_3_) δ 141.42 (t, *J* = 9.8 Hz), 138.06 (d, *J* = 11.78 Hz), 129.58 (dd, *J* = 30.8 Hz, *J* = 24.8 Hz), 129.50 (t, *J* = 263.7 Hz), 128.45, 128.36, 127.92, 127.81, 127.76, 127.70, 75.19, 73.66, 73.06, 71.18, 58.51(d, *J* = 4.36 Hz), 57.97 (t, *J* = 22.2 Hz); ^19^F NMR (282 MHz, CDCl_3_) δ −82.95 (ddd, *J* = 259.4 Hz, *J* = 15.2 Hz, *J* = 9.3 Hz, 1F), −97.6 (dd, *J* = 259.2 Hz, *J* = 9.9 Hz, 1F); IR (thin film) υ_max_ 2868, 1497, 1455, 1361, 1178, 1096, 1011, 738, 697 cm^-1^, MS(MALDI) *m/z* 401.1 (M + Na^+^). HRMS Calcd For C_21_H_21_O_2_F_2_ClNa: 401.1090; Found: 401.1088.

***trans-trans* dimer 8:** To a solution of **7** (29 mg, 0.077 mmol) in toluene (1.5 ml) Pd(dba)_2_ (40 mg, 0.069 mmol) was added. The reaction mixture was stirred at room temperature overnight. After removing the solvent in vacuo, the residue was purified carefully by flash chromatography (petroleum ether : ethyl acetate = 3 : 1) on silica gel to give **8** (27.5 mg) in 74% yield as a yellow solid (with 26% recovery of **7**); Crystals of **8** were obtained by slow diffusion of *n*-hexane in solutions of the complexes in dichloromethane at room temperature. Yellow powder: MP = 137 ^o^C, [α]D 26 = +136.53 (*c* 0.77, CHCl_3_); ^1^H NMR (300 MHz, CDCl_3_) δ 7.38-7.26 (m, 20H), 5.86 (s, 2H), 5.11 (s, 2H), 5.06 (s, 2H), 4.66-4.44 (m, 8H), 3.64-3.51 (m, 6H), 3.16-3.09 (m, 2H); ^13^C NMR (75.5 MHz, CDCl_3_) δ 137.84 (d, *J* = 12.84 Hz), 128.51, 128.41, 128.34, 127.88, 127.81, 127.77, 126.26 (dd, *J* = 155 Hz, *J* = 149 Hz); 105.42 (d, *J* = 3.6 Hz), 81.38, 76.30, 73.45, 73.16 (t, *J* = 19.3 Hz), 72.39, 69.97, 55.27(t, *J* = 14 Hz); ^19^F NMR (282 MHz, CDCl_3_) −90.75 (dd, *J* = 713 Hz, *J* = 245 Hz, 4F); IR (thin film) υ_max_ 1498, 1454, 1389, 1362, 1345, 1309, 1149, 1061, 1020, 738, 696 cm^-1^.

***syn-syn* dimer 9:** To a solution of **7** (16.2 mg, 0.043 mmol) in DMSO (2 ml), Pd(dba)_2_ (22.35 mg, 0.039 mmol) was added. The reaction mixture was stirred at room temperature overnight. The reaction mixture was quenched by water (3 ml) and extracted with dichloromethane. The combined organic phases were dried by Na_2_SO_4_. After removing the solvent in vacuo, the residue was purified carefully by flash chromatography (petroleum ether : ethyl acetate = 3 : 1) on silica gel to give **9** (17.9 mg) in 86% yield as a yellow sticky solid (with 14% recovery of **7**). Sticky solid: [α]D 26 = −141.93 (*c* 0.245, CHCl_3_); ^1^H NMR (300 MHz, CDCl_3_) δ 7.34-7.22 (m, 20H), 5.87 (s, 2H), 5.12 (s, 2H), 5.00 (s, 2H), 4.63 (s, 4H), 4.57 (q, *J* = 12Hz, 4H), 4.40 (s, 2H), 3.90-3.75 (m, 4H), 2.48-2.40 (m, 2H); ^13^C NMR (75.5 MHz, CDCl_3_) δ138.22 (d, *J* = 19.93 Hz), 128.42, 128.23, 127.75, 127.61, 127.44, 124.83 (t, *J* = 188 Hz), 105.23 (d, *J* = 6.64 Hz), 80.89, 77.75, 75.51 (t, *J* = 29 Hz), 73.49, 72.12, 68.47, 55.14 (dd, *J* = 20 Hz, *J* = 15 Hz); ^19^F NMR (282 MHz, CDCl_3_): −76.55 (dd, *J* = 244.8 Hz, *J* = 11.8 Hz, 2F), −105.7 (d, *J* = 250 Hz, 2F); IR (thin film) υ_max_ 1497, 1454, 1401, 1310, 1162, 1097, 1027, 740, 698 cm^-1^.

**3-Benzoyl-1-{(1*S*,5*R*)-5-[(*S*)-1,2-bis(benzyloxy)ethyl]-4,4-difluorocyclopent-2­enyl}-5-methylpyrimidine-2,4(1*H*,3*H*)-dione (5):** To a stirred solution of NaH (0.8 mg, 60% in oil) in anhydrous THF (1 mL), 3-benzoylthymine (4.6 mg) was added. The reaction mixture was stirred for 40 min at 40–45 °C and was then cooled to room temperature. Simultaneously, the mixture of PPh_3_ (5.4 mg, 0.02 mmol) and **8** (10 mg, 0.01 mmol) in anhydrous THF (1 mL) was stirred for 30 min at rt, in which the sodium salt of 3-benzoylthymine prepared beforehand was added. The reaction mixture was stirred at rt for 3 h. After removing the solvent in vacuo, the residue was purified carefully by flash chromatography (petroleum ether : ethyl acetate = 3 : 1) on silica gel to give **5** (27.5 mg) in 30% yield as a foam. [α]D 23 = −27.35 (*c* 1.07, CHCl_3_); ^1^H NMR (300 MHz, CDCl_3_) δ 7.93 (d, *J* = 9.0 Hz, 2H), 7.64 (t, *J* = 7.8 Hz, 1H), 7.45 (t, *J* = 8.1 Hz, 2H) , 7.34-7.28 (m, 10H), 6.97 (s, 1H), 6.41(d, *J* = 5.4 Hz, 1H), 6.28-6.25 (m, 1H), 5.95(t, *J* = 9.3 Hz, 1H), 4.78(dd, *J* = 87.3 Hz, *J* = 11.4 Hz, 2H), 4.48 (s, 2H), 3.78-3.72 (m, 2H), 3.58-3.52 (m, 1H), 3.24-3.09 (m, 1H), 1.74 (s, 3H); ^13^C NMR (100.7 MHz, CDCl_3_) δ 162.4, 150.46, 137.98, 137.78, 137.51, 137.36, 136.56, 134.99, 131.53, 130.44, 130.63 (t, *J* = 241.6 Hz), 129.22, 128.41, 128.31, 127.83, 127.75, 127.53, 127.37, 111.21, 74.84, 73.49, 72.41, 70.38, 57.81, 48.22 (t, *J* = 26.7 Hz), 12.21; ^19^F NMR (282 MHz, CDCl_3_) δ −93.09 (ddd, *J* = 265.0 Hz, *J* = 22.3 Hz, *J* = 7.3 Hz, 1F), −94.4 (dd, *J* = 248.7 Hz, *J* = 14.7 Hz, 1F); IR (thin film) υ_max_ 1751, 1702, 1656, 1260 cm^-1^, MS(MALDI) *m/z* 595.3 (M + Na^+^). HRMS Calcd For C_33_H_30_N_2_O_5_F_2_Na: 595.2015; Found: 595.2013


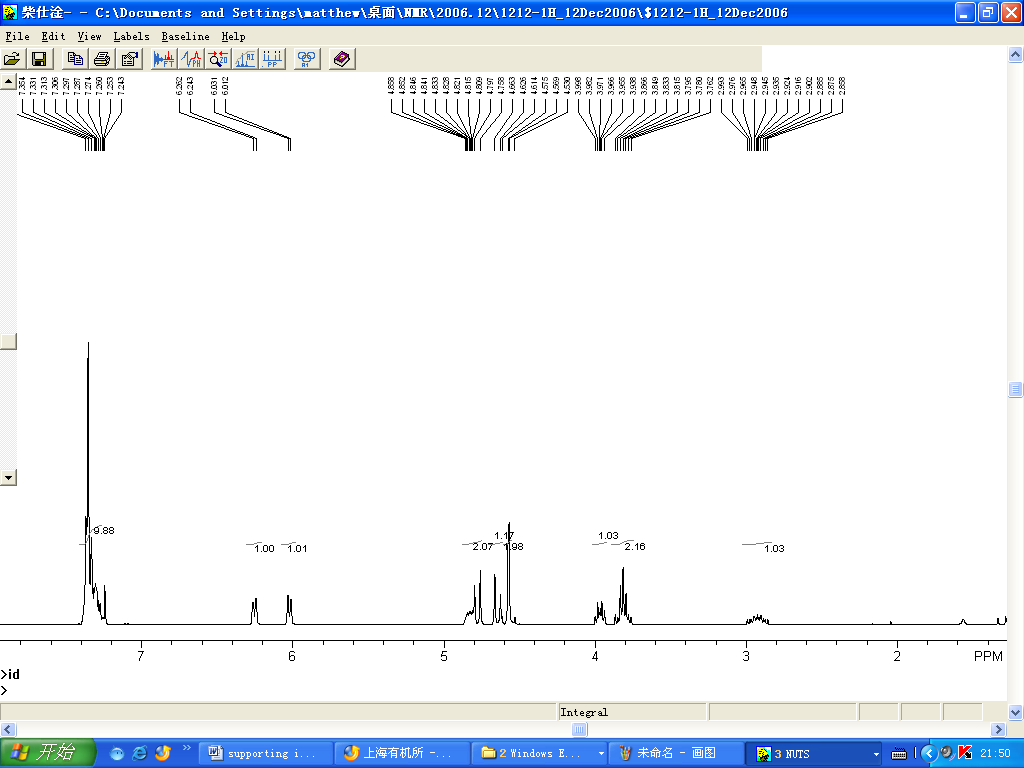


^1^H NMR Spectrum of compound **7**


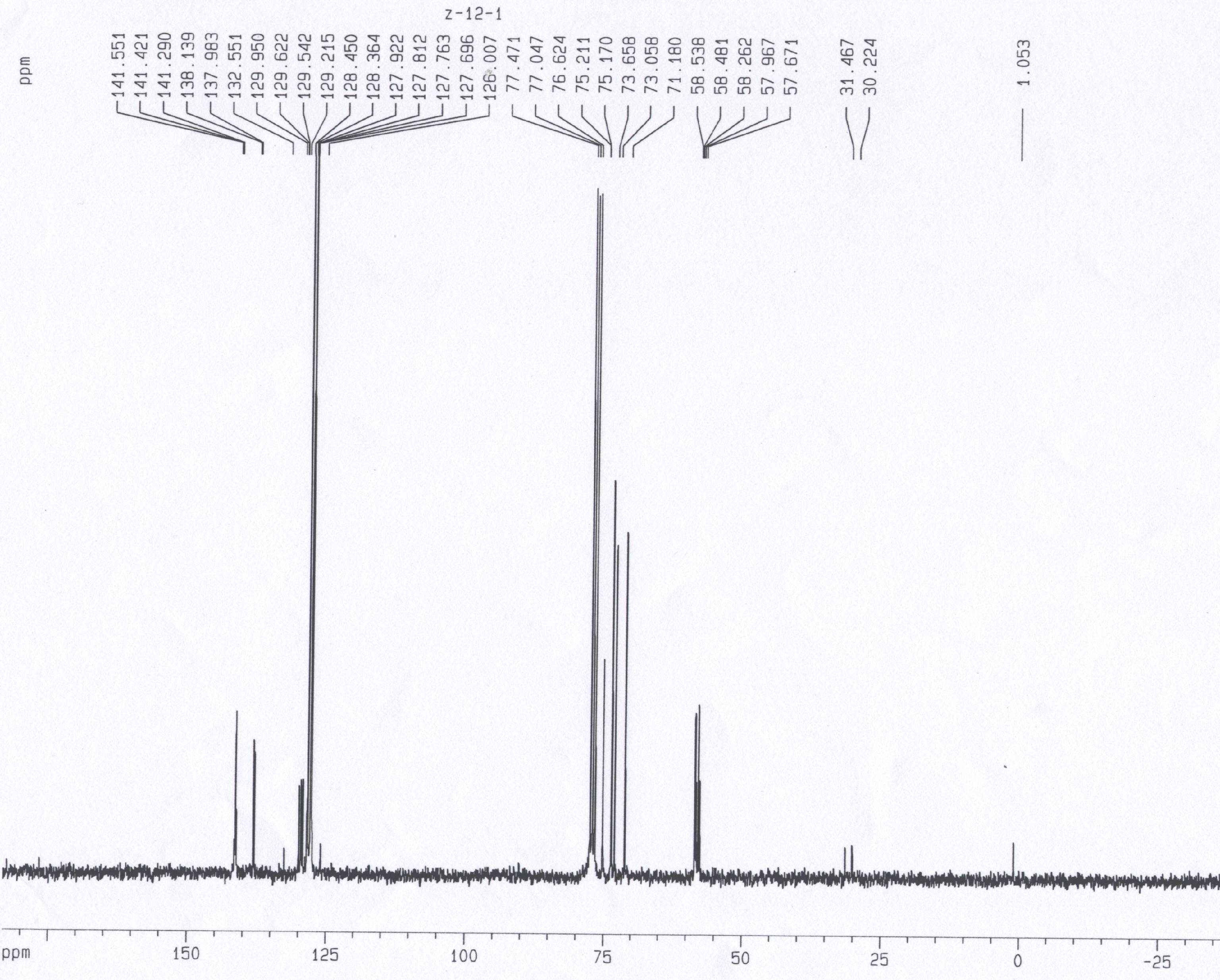


^13^C NMR Spectrum of compound **7**


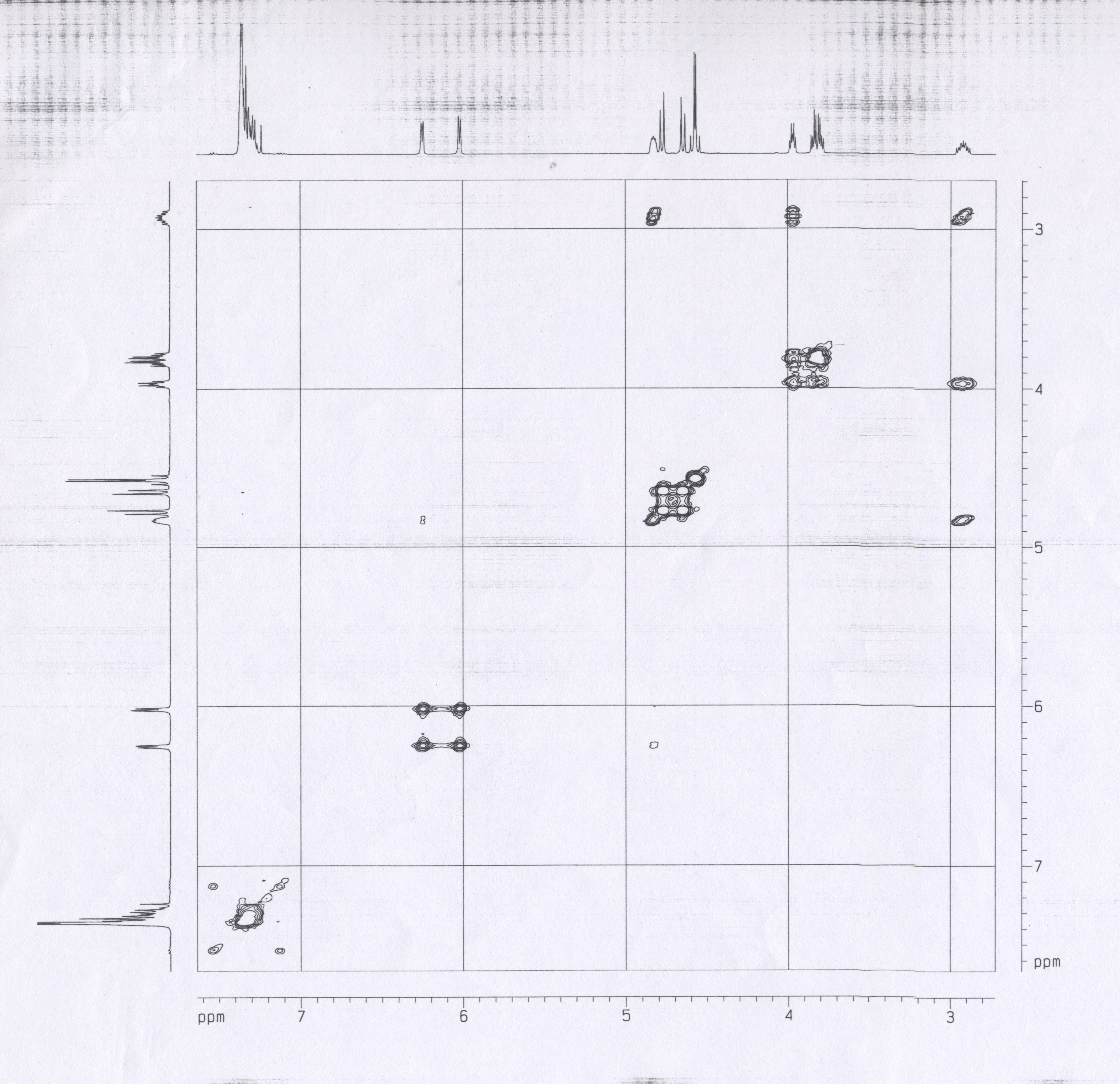


COSY Spectrum of compound **7**


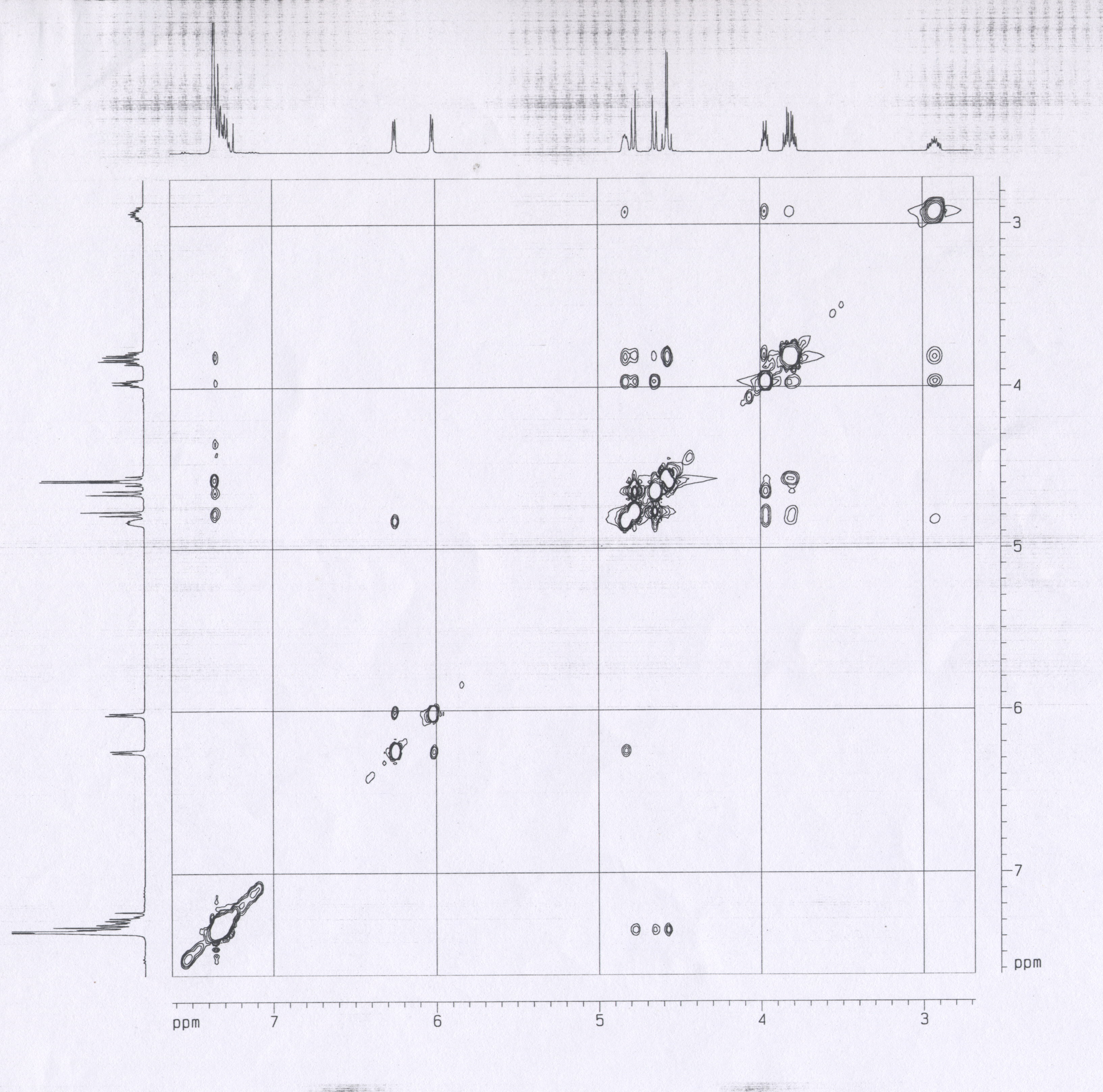


NOESY Spectrum of compound **7**


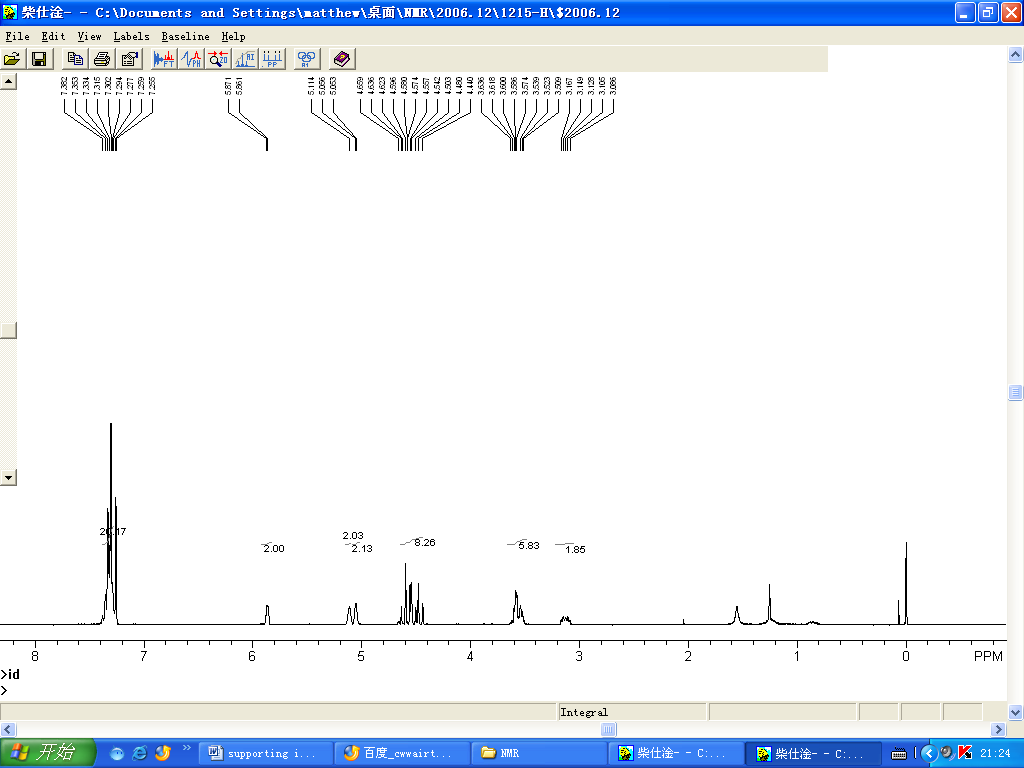


^1^H NMR Spectrum of compound **8**


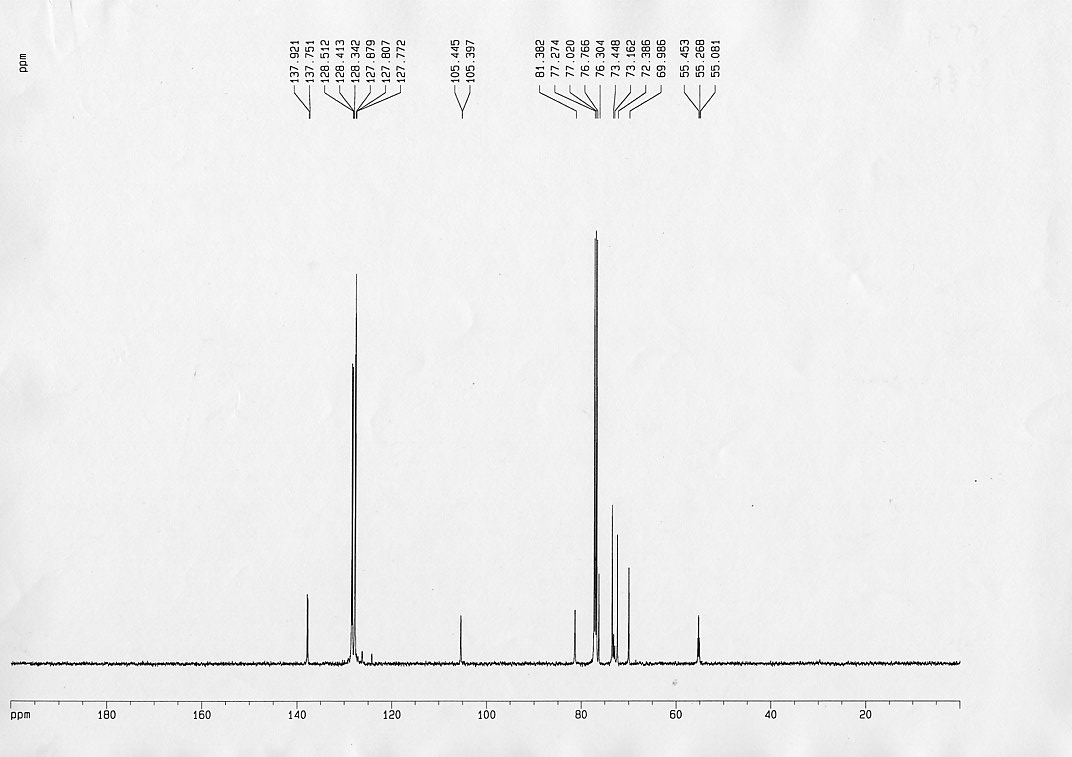


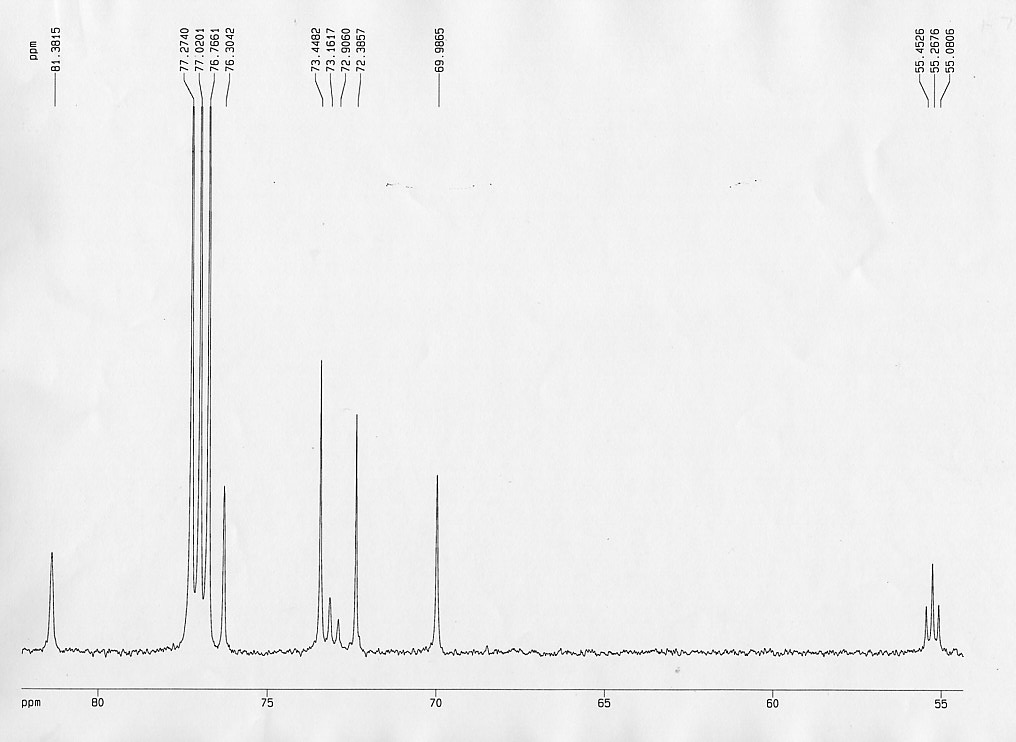


^13^C NMR Spectrum of compound **8**


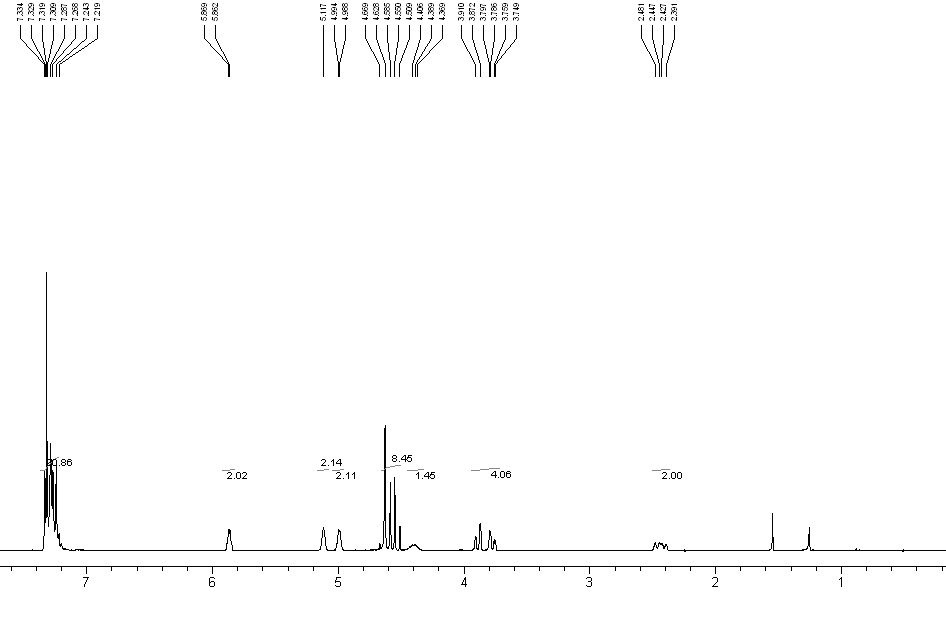


^1^H NMR Spectrum of compound **9**


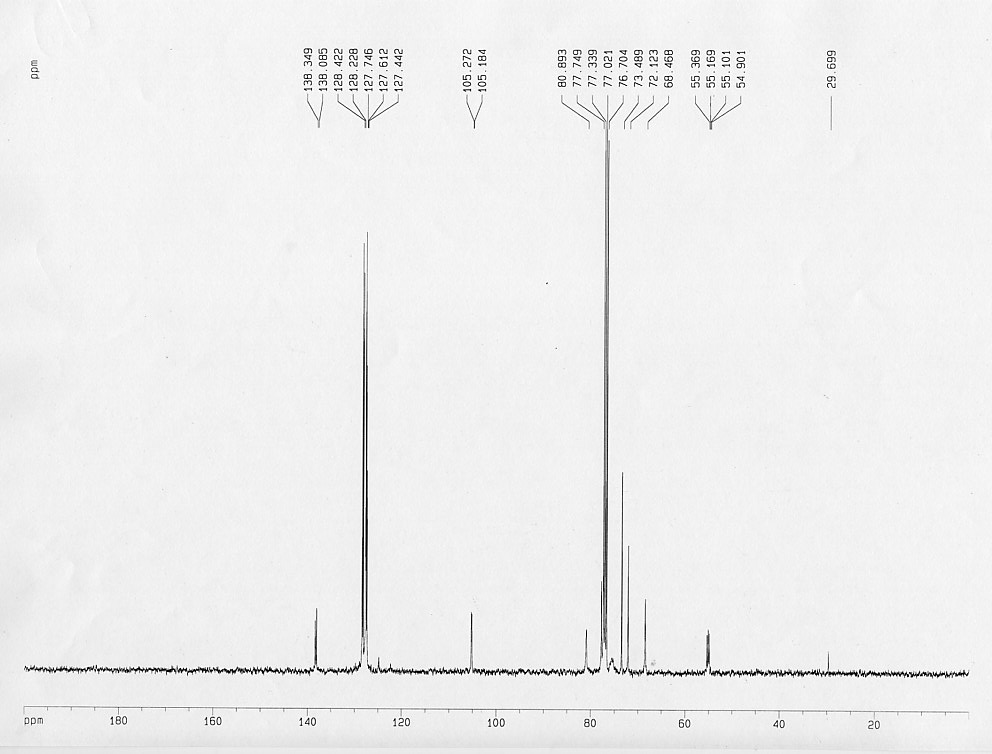


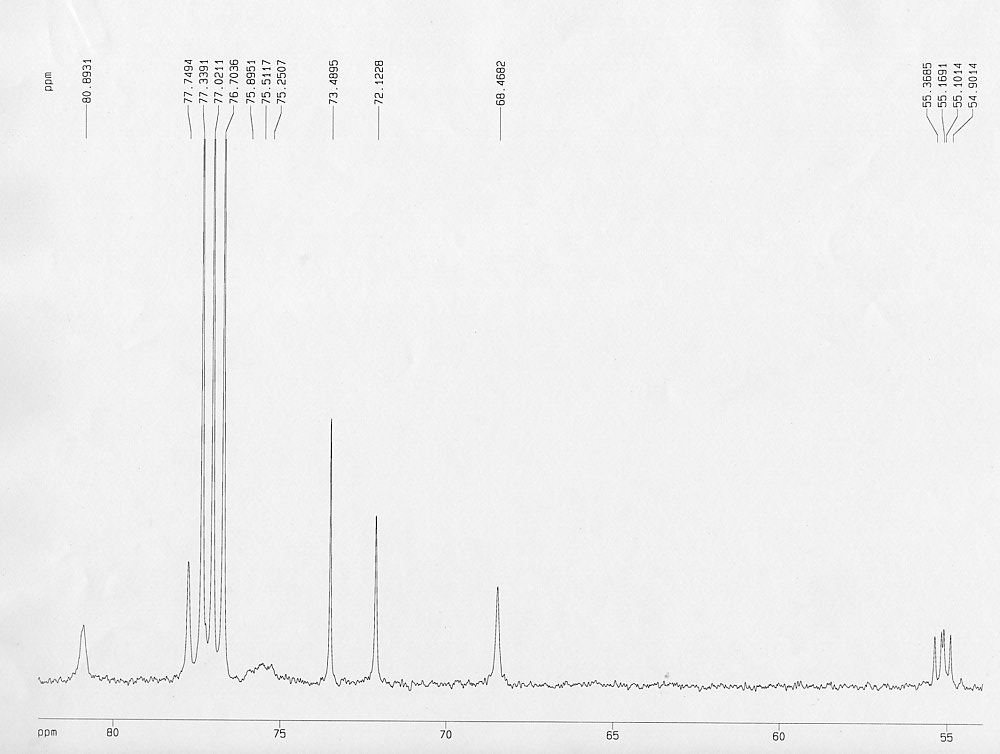


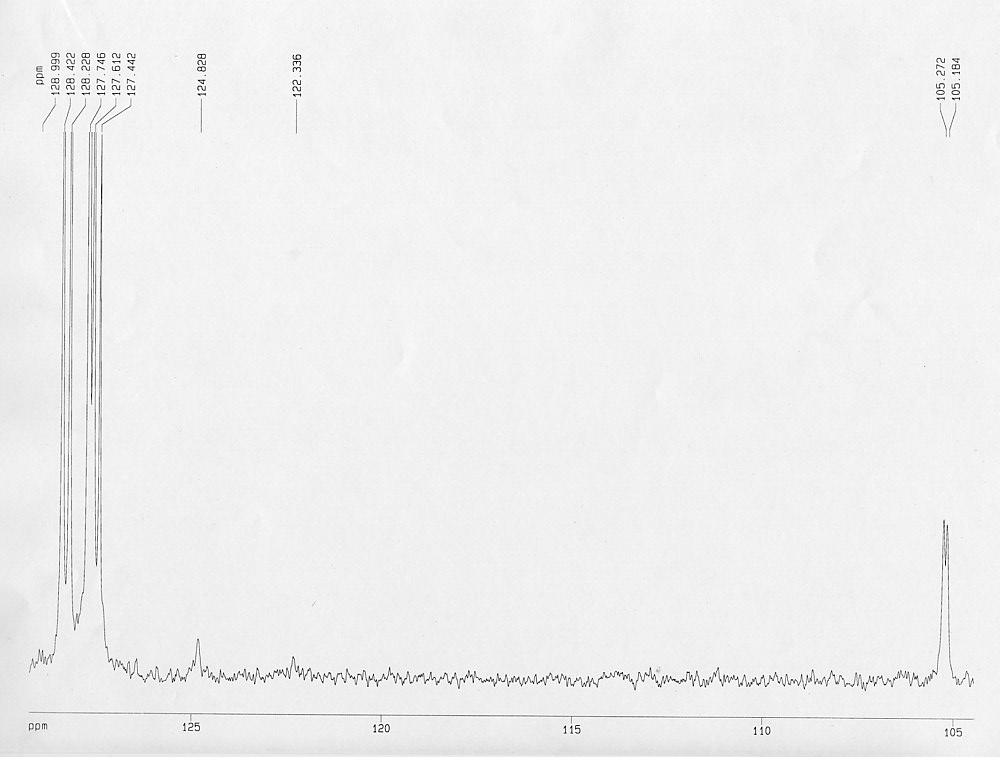


^13^C NMR Spectrum of compound **9**
